# Quality of referrals and adherence to guidelines for adult patients with minimal to moderate head injuries in a selection of Norwegian hospitals

**DOI:** 10.1007/s00068-024-02680-y

**Published:** 2025-01-24

**Authors:** Elin Kjelle, Ingrid Øfsti Brandsæter, Peter Mæhre Lauritzen, Eivind Richter Andersen, Jan Porthun, Bjørn Morten Hofmann

**Affiliations:** 1 Department of Health Sciences, Norwegian University of Science and Technology (NTNU), Postbox 191, Gjøvik, 2802 Norway; 2 Division of Radiology and Nuclear Medicine, Oslo University Hospital, Oslo universitetssykehus HF - Ullevål sykehus, 4956, Nydalen, Oslo, NO-0424 Norway; 3 Department of Life Sciences and Health, Faculty of Health Sciences, Oslo Metropolitan University, St. Olavs plass, P.O. Box 4, Oslo, NO-0130 Norway; 4 Centre of Medical Ethics, University of Oslo, 1130, Blindern, Oslo, 0318 Norway

**Keywords:** Computed tomography, Minor head injury, Justification, Referral quality

## Abstract

**Purpose:**

This study aimed to assess adherence to the Scandinavian guidelines, the justification of referrals, and the quality of referrals of patients with mild, minimal, and moderate head injuries in a selection of Norwegian hospitals.

**Methods:**

We collected 283 head CT referrals for head trauma patients at one hospital trust in Norway in 2022. The data included the patients’ sex, age, and the referral text. Six radiologists independently assessed all referrals using a registration form developed based on the Scandinavian guidelines for patients with mild, minimal, and moderate head injuries and general referral guidelines. Descriptive statistics was used to analyze data on adherence to guidelines, while Gwet’s AC1/2 was used to test the agreement between the raters.

**Results:**

This study found that 65% of referrals were assessed to be justified according to the guideline by at least one rater, while 17% were rated justified outside the guideline. In 52%, at least one rater required more information. There was good to moderate interrater agreement.

**Conclusions:**

Adherence to the Scandinavian guidelines and the quality of referrals of patients with mild, minimal, and moderate head injuries are low. Training and using S100B is recommended to improve the justification rate and quality of patient care.

## Introduction

Traumatic brain injury (TBI) is common worldwide. However, 70–95% of TBIs are mild [1–4]. Brain injuries can lead to long-term permanent sequelae, and the long-lasting treatment process financially burdens society; thus, diagnosing and treating these injuries efficiently is essential [5]. Computed Tomography (CT) is an effective imaging modality for diagnosing or ruling out brain injuries [6]. However, as a CT scan is a costly and limited resource that exposes the patient to ionizing radiation, head CT should only be used when the scan will provide information relevant to patient management and care [6]. Still, head CT is reported to be overused, especially in patients with minimal and mild head trauma with a low risk of TBI [3, 7–9]. Additionally, increased wait times due to unnecessary examinations may have severe consequences for other patients [10]. Inappropriate and unnecessary CT scans could undermine care quality, safety, and efficiency [11, 12].

In Norway, 10,000–15,000 people (0.3% of the population) suffer from brain injury annually. Falls and traffic accidents are the most common causes of TBI, and 30–50% of these patients are under the influence of alcohol [13]. Analyzing registry data from Norway from 2013 to 2021, an average of 126,801 head CTs (242 head CTs per 10,000 inhabitants) were performed annually, and a large patient group among these CT scans was head trauma patients.

Several guidelines have been developed and validated to ensure the appropriate use of head CTs in injuries, such as the Scandinavian guidelines, the Canadian CT head rule, the NICE guideline, the New Orleans criteria, and NEXUS-II [7, 14, 15]. The Scandinavian guidelines for initial management of minimal, mild, and moderate head injuries in adults, published in 2013, are the current guidelines in Norway [15]. The guideline recommends that a CT should be done in adult patients with a risk of mild and moderate TBI with Glasgow Coma Scale (GCS) ≤ 14, loss of consciousness, vomiting two or more times, anticoagulant therapy or coagulation disorders, clinical signs of depressed or basal skull fracture, post-traumatic seizures, or focal neurological deficits [15]. Further, the guidelines state that CT should not be done in adult patients as long as S100B is below 0.10 µg/l less than 6 h in risk of minimal and mild TBI with GCS 15 and without risk factors or after mild head injury with GCS 14 and no risk factors, or GCS 15 with loss of consciousness or vomiting two or more times and no other risk factors [15].

Adherence to guidelines is estimated to safely reduce the use of CT by 1/3 [6]. However, guidelines seem to have a low effect as head CT is repeatedly reported to be overused [6, 8, 9, 16–18]. Thus, the objective of this study was to assess the adherence to the Scandinavian guidelines, the justification of referrals, and the quality of referrals of patients with mild, minimal, and moderate head injuries in a selection of Norwegian hospitals.

We aimed to explore the following research questions:

1. To what extent do the referrals adhere to the Scandinavian guidelines for initial management of minimal, mild, and moderate head injuries in adults, and to what extent are they considered justified?
2. What is the interobserver agreement of justification?
3. Do head CT referrals for patients with minimal to moderate head injury include relevant and sufficient information to assess risk and justification?

## Methods

Referrals for head CT and patient background information were collected from one hospital trust in Norway. This hospital trust consists of 5 different local hospitals, all accepting acute head trauma patients. The hospital trust covers 340,000 people in a catchment area of 52,000 km^2^. In this hospital trust, 8,440 head CTs were performed in 2022.

The data collected included the patients’ sex, age, and referral text. Three radiographers working at the hospitals collected relevant information from all head CT referrals for adult patients (> 18 years) with mild to moderate head trauma conducted in 2022 and registered in the hospital trust’s Radiology information system (RIS). Inclusion and exclusion criteria are presented in Table 1.

**Table 1:** Inclusion and exclusion criteria for referrals

Include	Exclude
Patients with the clinical question of minimal or mild brain injury	Referrals coded as stroke
Patients referred due to head trauma or fall	Referrals coded as severe trauma
Referrals indicating intra-cranial bleeding	Contrast-enhanced head CT
	Patients doing scans of other body parts at the same appointment
	Age < 18 years
	Repeat head CTs for TBI

Any information identifying patients or personnel was removed. A data collection form was developed based on the Scandinavian Neurotrauma Committee guidelines for managing minimal, mild, and moderate head injury in adults [15]. The collection form included the referral, patient age and sex, and scoring categories. Each category had pre-defined answer options, as presented in Table 2. Each row also included a space for free text comments. The form was piloted with radiologists and amended after feedback. Even though GCS below nine is related to severe TBI, it is included in the scoring form to pick up possible severe TBI in the dataset.

**Table 2:** Scoring form with categories and possible answers

Tentative diagnosis is given	Sufficient and relevant case history is given	Information on time of injury is given	Sufficient and relevant clinical findings are given	Risk category based on guideline	GCS	S100B is given	Overall assessment
Yes No	Yes No	Yes No	Yes No	Moderate TBI Mild TBI-high risk Mild TBI-intermediate risk Mild TBI-low risk Minimal TBI Not enough information	< 9 9-13 14 15 Not given	Yes No	Justified within guideline Justified outside guideline Not justified Need more information

### Participants

Three consultant radiologists (A, B, C) and three radiology residents (D, E, F) from other hospital trusts in Norway independently assessed all referrals. Three worked at local hospitals, one at a regional hospital, and two at university hospitals. Radiology residents were included in addition to experienced radiologists, as residents are usually the ones assessing these referrals on-call at the hospital.

### Ethics

The Regional Committees for Medical and Health Research Ethics ​approved the anonymous data collection, ref no.268,688.

### Statistics

Microsoft^®^ Excel^®^ for Microsoft 365 MSO (Version 2207) was used for descriptive statistics. Kappa-statistics, Gwet´s AC1/2, was used to determine if there was an agreement between the raters [19] using Stata Statistical Software (Release 18), KAPPAETC [20]. The Kappa-statistic was interpreted as: <0.20 Poor, 0.21-0.40 Fair, 0.41-0.60 Moderate, 0.61-0.80 Good, 0.81 − 1 Very Good [21]. Since this study was not designed as a confirmatory study but to identify patterns, it was unnecessary to adjust tests for multiplicity. The significance tests used, therefore, have a descriptive character. A p-value < 0.05 was accounted statistically significant in all analyses.

#### Qualitative analysis of free text

Simple directed content analysis was applied to qualitatively analyze the free text comments [21].

## Results

After the inclusion and exclusion criteria assessment, 283 referral texts were enrolled for analysis. The participants (raters) rated the same 283 referrals. All the raters delivered a full dataset; hence, no data was missing.

### Justification

As shown in Table 3, a mean of 65% (range 46.6–77%) of the referrals was rated “Justified according to the guideline,” and 17% (range 12.7–23%) rated “Justified outside guideline.” Thus, 82% of the referrals were rated justified by one or more raters. In 41% (*n* = 115) of the referrals, all radiologists agreed that the referrals were justified in accordance with or outside the guidelines. Most of these referrals (*n* = 81) were rated “Justified according to guideline.” Further, a mean of 7% (range 0.4-9.2%) of the referrals was rated “Not justified,” while a mean of 11% (range 1.5–30.7%) of referrals was rated “Need more information.” Thus, 18% of the referrals were unjustified or of questionable justifiability. None of the referrals were assessed as “Not justified” by all radiologists.

**Table 3:** Number and percentage of justification assessments per radiologist

	Radiologist						
	A	B	C	D	E	F	Mean
	n (%)	n (%)	n (%)	n (%)	n (%)	n (%)	n (%)
Justified according to guideline	207 (73.1)	189 (66.8)	187 (66.1)	132 (46.6)	178 (62.9)	218 (77.0)	185.2 (65)
Justified outside guideline	44 (15.5)	65 (23.0)	36 (12.7)	63 (22.3)	37 (13.1)	39 (13.8)	47.3 (17)
Not justified	22 (7.8)	25 (8.8)	14 (4.9)	1 (0.4)	26 (9.2)	21 (7.4)	18.2 (7)
Need more information	10 (3.5)	4 (1.5)	46 (16.3)	87 (30.7)	42 (14.8)	5 (1.8)	32.3 (11)

**Fig. 1:**
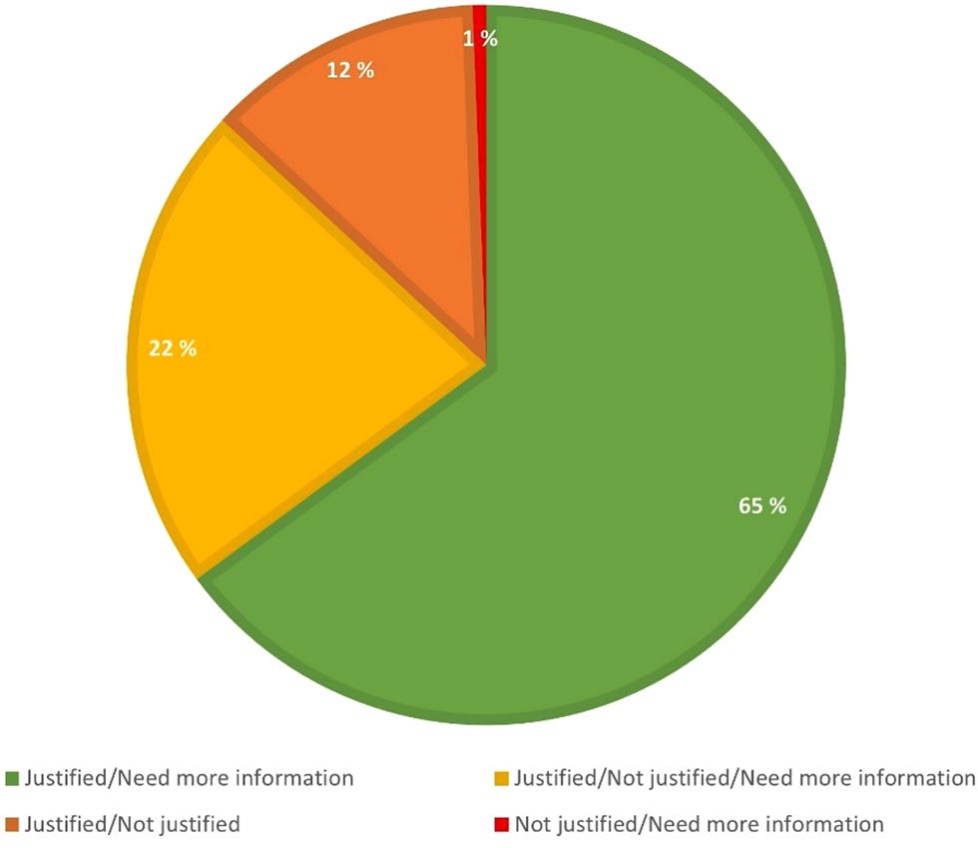
Overview of how the 168 referrals where the radiologists disagreed were scored

The raters disagreed on 168 referrals (59%). Figure 1 shows how referrals with disagreement were rated. In 65% (*n* = 109) of referrals without a uniform rating, the disagreement was a combination of “Justified” and “Need more information.” In 22% (*n* = 37) of these referrals, the assessment was a combination of three ratings, “Justified,” “Need more information,” or “Not justified.” While 12% (*n* = 21) of these referrals were assessed as “Justified” or “Not justified,” only 1% of the referrals were assessed as “Not justified” or “Need more information.”

## Interrater reliability

The overall interrater agreement was moderate κ = 0.57 (95% CI, 0.53 to 0.62), *p* <.0001. In the subgroup of the three consultant radiologists (A-C), the interrater agreement was good, κ = 0.70 (95% CI, 0.65 to 0.76), *p* <.0001. While in the subgroup of the three resident radiologists (D-F), the interrater agreement was moderate, κ = 0.43 (95% CI, 0.37 to 0.49), *p* <.0001. Table 4 shows the individual interrater agreement between consultant and resident radiologists, showing that rater D has a fair interrater agreement. In contrast, rater E and F have good interrater reliability with the consultant radiologists.

**Table 4:** Interrater reliability between consultant radiologists and resident radiologists

	Resident radiologists		
	D	E	F
Consultant radiologists	Gwet´s AC1/2 (95% CI)	Gwet´s AC1/2 (95%CI)	Gwet´s AC1/2 (95%CI)
A	0.39 (0.31-0.47)	0.67 (0.60-0.74)	0.67 (0.60-0.73)
B	0.43 (0.35-0.51)	0.71 (0.64-0.77)	0.67 (0.60-0.73)
C	0.36 (0.28-0.44)	0.69 (0.62-0.76)	0.60 (0.53-0.67)

Confidence interval (CI)

## Information in the referral text

In 52% (*n* = 147) of the referrals, the overall assessment of justification by one or more radiologists was “Need more information.” In 112 of these referrals, only one radiologist concluded that more information was needed.

**Fig. 2:**
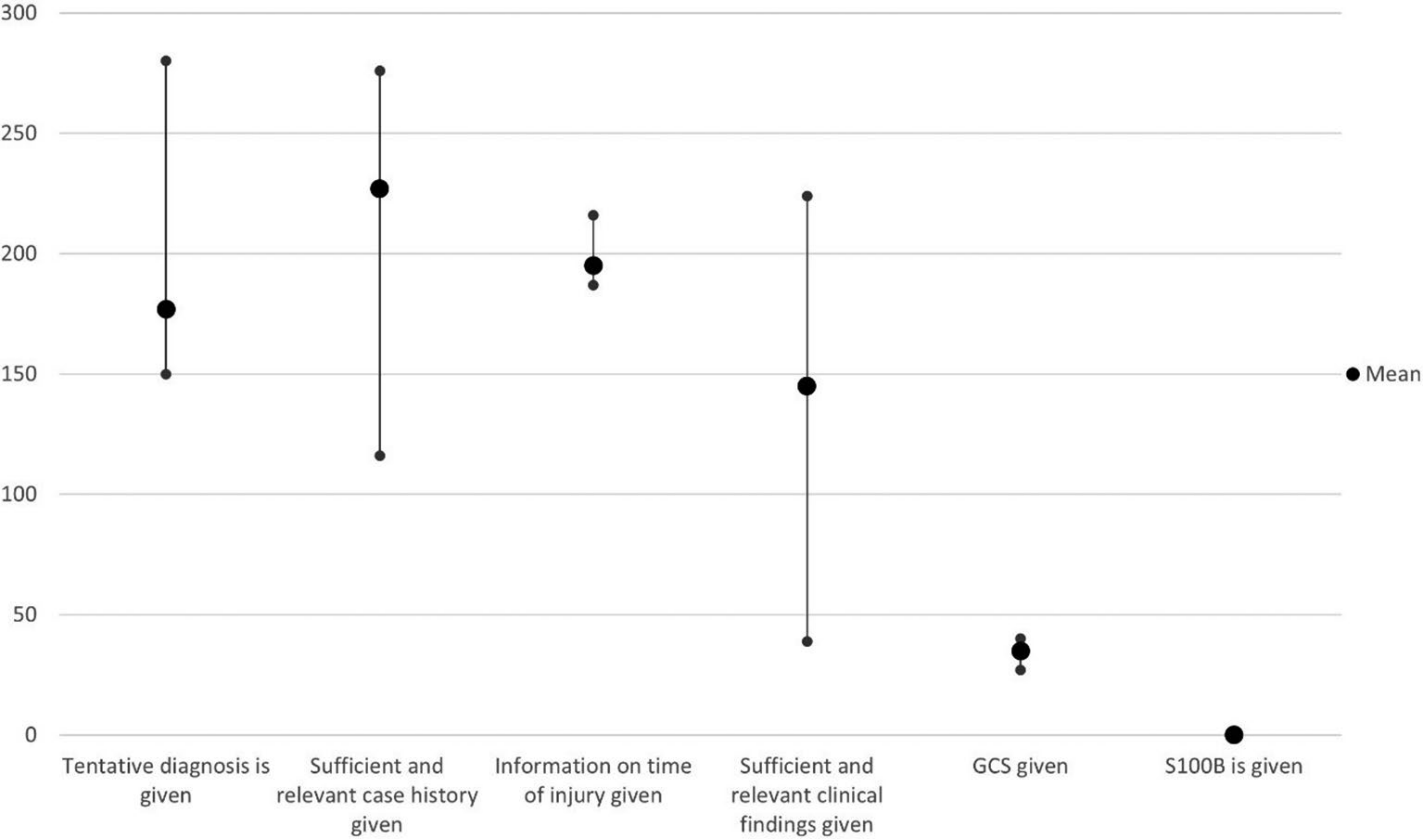
The mean, minimum, and maximum number of the 283 referrals rated “Yes” in the referral information categories and where a GCS score was provided

As shown in Fig. 2, when looking at the individual rater’s scoring, less than half of the referrals lacked information in the categories GCS (mean 35) and Sufficient and relevant clinical findings (mean 145). The raters mostly agreed in their rating of the GCS and time of injury, hence the short error bars. At the same time, the other categories had large variations (long error bars) in rating whether sufficient information was provided. None of the referrals included information about the S100B score, indicating that the included hospitals do not use the S100B blood test in TBI patients, even though the use of S100B is recommended in the guideline.

From the information in the referrals, the radiologists assessed the risk category. A mean of 10.7% of the referrals did not have enough information to define the risk category. Further, 13.3% were in the minimal category, where CT was not recommended. 37.3% of referrals were categorized as moderate or mild in the high-risk category that, according to the guideline, should have a CT. 22.6% of the referrals were in the mild, mid-risk category where the guideline suggests CT or observation. 16.2% of the referrals were in the mild, low-risk category, where S100B is recommended within 6 h after injury.

### Free text comments

Five raters used the free text option in the registration form, totaling 358 insertions. The mean number of insertions was 60, ranging from 0 to 129. Six categories were identified: Different examination, Justification, Referral quality, Lack of information, Assumptions, and About the form. An overview of categories, the number of comments, and an example from each category are provided in Table 5. Most comments were given in the categories Assumptions (*n* = 156) and Justification (*n* = 124).

**Table 5:** Categories of free text comments given in the registration form, with an example of typical statements in each

Category	Number of insertions	Example
Assumptions	156	Supposed the time of injury was the same day
Justification	124	Liver failure qualifies as coagulation disturbances
Lack of information	51	No information about neurological findings
“About the form”	10	I did not fill in GCS when it was not specified, even though one can reason up to it
Referral quality	9	Perfect referral
Different examination	8	Should refer to CT of the temporal bone or MRI instead

GCS: Glasgow coma scale, CT: Computed tomography, MRI: Magnetic resonance imaging

The Assumptions category included the raters’ assumptions when lacking information in the referral. The most frequently reported assumptions were about the time of injury and the clinical question of the referral (bleeding or fracture). The Justification category included reasons why radiologists assessed examinations as justified according to or outside guidelines (for example, clinical issues, risk assessments, or legal considerations when patients were exposed to violence). Regarding the lack of information, raters commented on the need for more details about the patient and clinical issues, such as patient history, medication, results from clinical assessments, and neurological symptoms. In the category About the form, general comments about how the raters chose to interpret the questions, the referral text, and how to fill out the form. The comments in Different examinations indicated that another or additional tests to head CT should be performed, e.g., a full trauma scan or brain MRI.

## Discussion

In this study, a mean of 82% of the referrals were found justified by one or more raters. The radiologists agreed that 41% of the referrals were justified, while no referrals got a uniform classification as not justified. When there was disagreement, “Justified” was most often combined with “Need more information” (65%). This study showed moderate interrater agreement overall with a good interrater agreement in justification rating amongst the consultant radiologists. The moderate agreement was mainly caused by one rater more often wanting more information than the other raters. Differences in justification practice in CT and, thus, how referrals are assessed have been shown in earlier research [22].

Radiologists’ assessing referrals often lack essential information for justification assessment, and a good quality referral providing this information is necessary for a high-quality imaging service [23]. In this study, the referrals often lacked information on the patient’s GCS score and other relevant clinical information. This information would be essential as the quality and outcome of image assessment strongly depend on the amount and content of the referral information [23]. In addition, the hospital trust in this study does not seem to use the S100B test, as none of the referrals provided details on this test in the referral text. The S100B can help predict which patients with mild head injury and low risk have good use of a CT scan within 6 h after injury [1, 4, 24, 25]. 16% of the referrals were rated in this category. Thus, if used according to the guideline [15], there could be a 16% reduction in head CTs. Further, 13% of the referrals were rated in the minimal category, and these patients should, according to the guidelines, not have a CT.

In addition to the moderate interrater agreement showing a variation in rating, there was also a large variation in the rating on whether there was a tentative diagnosis in the referral or if there was enough clinical information and case history. Thus, the assessment seems based on subjective judgments or raters making different assumptions based on the referral text. The subjective assessments and quality of guidelines for the referrer and radiologists might be a reason for the variation in the rate of unnecessary and inappropriate imaging seen internationally [6, 8, 16–18].

The assumptions made by the raters in this study were addressed in the free text comment section of the form by several of the raters. For example, one frequently used comment was, “*Based on the information in the referral*,* I interpret it as implicit that the tentative diagnosis is bleeding and/or fracture.”* The free text comments revealed that the raters made assumptions that might influence the results. One frequently free text comment was about the time of injury, where rater assumptions yielded if the examination was justified according to or “outside” the guidelines, e.g., “*I suppose the time of injury is > 24 hours and choose outside guideline”.* However, despite variations in assumptions yielding variations in the agreement of guideline adherence, the conclusion was most often the same.

International research has shown an over-use of head CT in patients with TBI and with a low incidence of relevant findings (2–7%) [8], even with clinical guidelines in use [9]. Thus, the unnecessary use of head CT might be even higher than suggested in this study. Further, in the last decades, the use of head CT has increased, while at the same time, the diagnostic yield has decreased [9]. This suggests training and possibly a need for guideline revision to avoid the unnecessary use of ionizing radiation and more sustainably use CT and imaging capacity.

### Strengths and limitations

This study thoroughly assessed guideline adherence, justification of the referrals, and assessment of the information in head CT referrals for head injury. However, there are weaknesses to address. The raters did not have access to the exact incidence time of all patients as this was a retrospective study. Some of the referrals stated that the injury occurred on a specific date. However, the rater did not know the date of referral. Thus, the time that elapsed since the incident could have been assumed differently in some referrals than in a prospective setting. Further, the raters could not access the patient’s medical records. In a clinical setting, the radiologist might have the opportunity to check the record for additional information or to communicate with the referring physician. Thus, the *Need more information* rate might have been lower in a clinical setting. The study did not assess the radiological outcomes of referrals or their clinical consequences. While beyond the scope of this study, these are related and important topics for future research. In addition, we would recommend training for referrers on the Scandinavian guidelines and the use of S100B in these hospitals, which should be assessed in further follow-up. As earlier research shows, overusing head CT in these patients is a problem in several countries [8]. Thus, it is likely that similar findings apply in other European countries, even if the guidelines and referral vetting procedures are different [22].

## Conclusions

In this study, 65% of the referrals were rated “Justified according to guidelines,” 17% were rated justified outside guidelines, and 82% rated justified overall by one or more raters. Overall, 7–18% of the examination could possibly have been avoided. Further, according to the risk category rating, up to 30% of referrals could have been avoided. A moderate interrater agreement pointed to a subjectivity in referral assessment. More than half of the referrals lack relevant information. Lack of information might lead to unnecessary imaging and reduced quality, safety, and efficiency in the health services.

## Data Availability

The datasets used and/or analyzed during the current study are available from the corresponding author on reasonable request.

## Abbreviations

CI: Confidence interval
GCS: Glasgow Coma Scale
RIS: Radiology information system
TBI: Traumatic brain injury
